# Use of Transcranial Direct Current Stimulation via Flow FL-100 Within Community Mental Health Services (CMHT) for Patients with Depression

**DOI:** 10.1192/bjo.2025.10205

**Published:** 2025-06-20

**Authors:** Faquiha Muhammad, Mohammed Al-Dabbagh, Agastya Nayar, Chris Griffiths

**Affiliations:** NHFT, Northampton, United Kingdom

## Abstract

**Aims:** The study evaluates the use of transcranial direct current stimulation (tDCS) via a portable device, called **Flow FL-100**, within Community Mental Health Services (CMHT) for patients with depression. This service targets individuals who either did not benefit from medication or sought alternatives to it.

Flow is a tDCS treatment for depression that patients can use at home. It is safe, well-tolerated, and free from the side effects commonly associated with antidepressants. Backed by over 30 years of research, tDCS has shown significant improvements in depressive symptoms, with high clinical response rates. Recent studies show remission rates of up to 45% with Flow. The treatment is CE-marked for use in Europe, and a recent NICE briefing (2023) highlights its efficacy and safety. The evaluation also references positive results from randomised controlled trials (RCTs), showing Flow leads to better outcomes than placebo stimulation.

**Methods:** This service evaluation assessed the use of tDCS for treating depression in Community Mental Health Teams (CMHT) for patients who hadn’t responded well to medication or wanted an alternative. After a clinical interview and assessment, eligible patients were offered the treatment. Outcome data was collected at baseline and again after 6 weeks, using the Montgomery– Åsberg Depression Rating Scale (MADRS). The treatment involved patients self-administering tDCS for 30 minutes, five times a week for three weeks, then three times a week for three more weeks, with option of continuing as needed. The “Flow” system also includes a lifestyle training app and symptom tracking, allowing patients and clinicians to monitor progress online.

The study used an open-label design without a control group, with 20 participants (12 males, 8 females), of whom 16 shared their progress on the online platform and were included in the analysis.

**Results:** We analysed 16 data sets, which showed the following **Results:** the average MADRS score at the initial assessment was 32. By week 6, 82% (12/16) of participants had improved on the MADRS scale, with 44% (7/16) demonstrating clinically significant improvement, marked by a reduction of more than 25% in their MADRS score.

**Conclusion:** These results indicate that tDCS portable device “Flow” treatment is a promising and valuable intervention for treating depression in adults in CMHT service.

